# The Role of Brown Adipose Tissue and Energy Metabolism in Mammalian Thermoregulation during the Perinatal Period

**DOI:** 10.3390/ani13132173

**Published:** 2023-07-01

**Authors:** Cécile Bienboire-Frosini, Dehua Wang, Míriam Marcet-Rius, Dina Villanueva-García, Angelo Gazzano, Adriana Domínguez-Oliva, Adriana Olmos-Hernández, Ismael Hernández-Ávalos, Karina Lezama-García, Antonio Verduzco-Mendoza, Jocelyn Gómez-Prado, Daniel Mota-Rojas

**Affiliations:** 1 Department of Molecular Biology and Chemical Communication, Research Institute in Semiochemistry and Applied Ethology (IRSEA), 84400 Apt, France; 2 School of Life Sciences, Shandong University, Qingdao 266237, China; 3 Animal Behaviour and Welfare Department, Research Institute in Semiochemistry and Applied Ethology (IRSEA), 84400 Apt, France; 4 Division of Neonatology, Hospital Infantil de México Federico Gómez, Mexico City 06720, Mexico; 5 Department of Veterinary Sciences, University of Pisa, 56124 Pisa, Italy; 6 Neurophysiology, Behavior and Animal Welfare Assessment, DPAA, Universidad Autónoma Metropolitana, Xochimilco Campus, Mexico City 04960, Mexico; 7 Division of Biotechnology-Bioterio and Experimental Surgery, Instituto Nacional de Rehabilitación Luis Guillermo Ibarra Ibarra (INR-LGII), Mexico City 14389, Mexico; 8 Clinical Pharmacology and Veterinary Anesthesia, Facultad de Estudios Superiores Cuautitlán, Universidad Nacional Autónoma de México (UNAM), Cuautitlán Izcalli 54714, Mexico

**Keywords:** brown adipose tissue, mammals, altricial, precocial, hypothermia, thermostability, neonate

## Abstract

**Simple Summary:**

Brown adipose tissue (BAT) is a thermogenic tissue that greatly contributes to preventing neonatal hypothermia by activating biochemical and endocrine processes because of cold stress. The presence of uncoupling proteins and adrenergic receptors in the brown adipocyte initiates the metabolic pathway for heat production. However, the presence and absence of BAT, as well as its activation, location, and the degree of thermogenic response, are traits that depend on intrinsic and extrinsic factors of mammals. The present review aims to discuss the neuromodulation mechanisms of thermoregulation and the importance of BAT, emphasizing the analysis of the biochemical, physiological, and genetic factors that determine the distribution, amount, and efficiency of this energy resource in newborns of different species.

**Abstract:**

Hypothermia is one of the most common causes of mortality in neonates, and it could be developed after birth because the uterus temperature is more elevated than the extrauterine temperature. Neonates use diverse mechanisms to thermoregulate, such as shivering and non-shivering thermogenesis. These strategies can be more efficient in some species, but not in others, i.e., altricials, which have the greatest difficulty with achieving thermoneutrality. In addition, there are anatomical and neurological differences in mammals, which may present different distributions and amounts of brown fat. This article aims to discuss the neuromodulation mechanisms of thermoregulation and the importance of brown fat in the thermogenesis of newborn mammals, emphasizing the analysis of the biochemical, physiological, and genetic factors that determine the distribution, amount, and efficiency of this energy resource in newborns of different species. It has been concluded that is vital to understand and minimize hypothermia causes in newborns, which is one of the main causes of mortality in neonates. This would be beneficial for both animals and producers.

## 1. Introduction

Parturition is considered an abrupt process that submits mammals to a wide variety of challenges. The challenge of adequately thermoregulating is enhanced because after staying in an environment where the temperature remains regulated and relatively constant, as is the uterus, neonates face an environment with a temperature at least 10 °C lower than in utero [1,2,3]. This change is potentially stressful even in precocial species such as lambs, who, regardless of the thermoregulatory advantages (e.g., adipose tissue deposits, motor ability to stand up immediately after birth) are still exposed to body temperature drops. This could be attributed to newborns being vulnerable to thermal changes that could cause hypothermia from birth until 36 h of life, and because they may experience excessive heat loss and depletion of their energy reserves [1,4,5,6]. In fact, in preterm infants, the mortality rate can increase markedly, even with short periods of hypothermia [7,8]. This shows how fundamental thermoregulation and thermogenesis are for the survival of the animals. Moreover, having sufficient energy reserves, mainly through the consumption of colostrum and milk, is an important source of thermogenesis fuel [1,9,10]. The main defense mechanisms against the cold in newborns include: (i) vasoconstriction with the objective to maintain heat in the core of the body and minimize heat loss [11]; (ii) non-shivering thermogenesis (NST), which involves the metabolism of brown adipose tissue (BAT); and (iii) shivering thermogenesis [1]. Nonetheless, the contribution of shivering to the thermoregulation of neonates has been questioned [12], since in the vast majority of species it has been seen that fat is the main source of energy used to cover their high metabolic rates and because of the relative immaturity of neonates’ musculature to produce heat by shivering [1,12]. However, in species such as the pig, the content of adipose tissue of any type in neonates is small (1.5%), which has led to ruling out the development of NST [13]. However, recent studies mention the existence of an NST mechanism that does not involve BAT [14].

The activation of NST using BAT is regulated by the central nervous system (CNS) when responding to peripheral thermal sensory inputs. Transient receptor potential (TRP) cation channels—particularly TRPM8 and TRPA1 to detect cold—[15] receive signals associated with changes in the environmental temperature [16]. For this reason, it is relevant to know the thermoregulation procedures used by various species, as well as the factors that can influence their development, since all this knowledge can be useful for the creation of strategies that ensure the welfare and survival of newborns, an aspect of great interest for production systems that present significant economic losses due to neonatal mortality of up to 50% [3,4,5]. Thus, this article aims to discuss the neuromodulation mechanisms of thermoregulation and the importance of BAT in the thermogenesis of newborn mammals, emphasizing the analysis of the biochemical, physiological, and genetic factors that determine the distribution, amount, and efficiency of this energy resource in newborns of different species.

## 2. Uterine Thermoregulation of the Fetus

The proper functioning of cellular processes in the body during the gestation period is essential because the mother provides the energy resources for the physical development and physiological maintenance of the fetus through maternal–fetal communication [17]. Uterine temperature is 0.5 °C above the dam’s, and this interchange modifies fetus temperature. This is obtained through the placental surface (2.1 watts per kg) and the umbilical circulation [18] to afterward dissipate heat through the placental surface and the fetal membranes [19].

Different studies have verified that fetuses manage to warm up through their metabolic processes. Power et al. [20] found that fetal sheep can produce about 3.3 watts per kilogram of fetal tissue, which is equivalent to 47 calories per minute, and about twice the heat that an adult can produce per unit of body weight. Fetal oxygen consumption after occluding the umbilical cord is 6.7 mL/kg/min, 1.5 times higher than that of adults. This demonstrates that the fetal basal metabolic rate is higher, resulting in significant heat production that causes fetuses to have a temperature 0.3–0.5 °C higher than the mother, even when she develops a fever [18,21]. It must be emphasized that even non–pathological events, for example, exercise, lead to umbilical cord occlusion [12,22].

An example of heat dispersion in the fetus is detailed in the study by Gilbert et al. [23], who detected that 85% of the heat generated by a fetal lamb was transferred to the mother through the circulation and the last 15% was transferred from her skin to the amniotic sac to reach the uterine wall later, maintaining a balance in the production and loss of heat [8]. However, heat transfer through maternal–fetal communication can be affected by alterations in umbilical blood flow, triggering an increase in fetal temperature, which has been reported in fetal baboons and fetal lambs that experienced partial occlusion or total, respectively, of the umbilical cord [24,25].

This has been verified with the use of tele-thermography, with which was detected that the surface temperature of infant newborns was higher if they had a coiled umbilical cord [26], indicating that fetal temperature changes rapidly because of disturbances in the umbilical blood flow that do not allow it to dissipate heat. Something similar to what was previously described happens when the mother experiences a 2.5 °C increase in temperature because, due to the initiation of a cutaneous vasodilation response in the dam, blood flow to the umbilical cord decreases significantly, which would explain the reason why, even in the face of fever, the fetus has a higher temperature than the mother [27].

Thus, to survive the extrauterine environment that represents a thermal challenge, fetuses must accumulate BAT before parturition, so premature newborns have a reduced capacity to initiate BAT thermogenesis to face the cold from the extrauterine environment [28]. This has at least been verified in infants born before reaching 30 weeks, in whom NST activity could not be detected on the dorsal surface with thermography [29]. Similarly, preterm lambs have been reported to show reduced rectal temperature, low thyroid hormone concentrations, and altered adrenal cortex activity compared with those born at full term [30]. It also has been reported that lambs born by caesarean section, in addition to presenting low concentrations of thyroid hormone in plasma, have a reduced content of norepinephrine compared to those that were born vaginally [31]. It should be noted that lambs born by cesarean section are not uncovered by the usual peaks of corticosteroids and catecholamines that are necessary for the physiological maturation of the newborn [6]. Besides, it has been described that the victorious activation of BAT, also known as thermogenesis without a tremor, depends on the grade of maturation at birth that different species possess [32] (or even within the same species [1]). For this reason, these individuals with immature thermoregulation must be kept in adequate thermal conditions [12].

## 3. Newborn Thermoregulation

During pregnancy, heat is transferred to the fetus through the placental blood vessels and the uterus; therefore, fetal temperature depends on the mother until birth. Consequently, when the mammalian neonate is born, it faces various challenges, including exposure to an environment generally below intrauterine temperature, significant evaporative heat loss [2], and adjustments to cardiorespiratory function. Thus, effective thermoregulation mechanisms are activated during the neonatal period, triggering specific thermogenic responses a few minutes after birth [2]. This continues for several hours due to the high risk that compromises the survival of the neonate [33]. Individual characteristics (e.g., birth weight and vitality) [34], energy reserves, and colostrum intake available at birth add to adequate oxygenation processes to avoid hypoxemia and a deficit in heat production.

In response to cold stress that occurs during and after birth, two mechanisms are activated to generate heat: the first is an increase in cellular metabolic activity and heat production by shivering thermogenesis, and the other is NST, characterized by lipolysis in BAT [35]. The low environmental temperatures perceived by the neonate set the tone for the initiation of negative feedback processes corresponding to the hypothalamic–pituitary–adrenal (HPA) axis activation. This is another neuroendocrine mechanism that is also involved in both central and peripheral temperature control. Its activation leads to an increase in catecholamines and cortisol in the blood. In addition to the above, to continue with thermal homeostasis, mechanisms for maintaining temperature are added, such as dermal vasomotor control, piloerection, shivering, BAT activation, and huddling. The previously mentioned mechanisms aim to avoid hypothermia that would put the life of the newborn at risk [34]. It should be noted that these mechanisms depend on factors such as parental care; litter size; anatomical, physiological, and behavioral characteristics of the species; sensorimotor maturity; and environmental characteristics present at birth, to name a few [36,37,38]. In this sense, in most species, the resistance to cooling can be compromised. It is known that altricial species such as rabbits, rats, and mice are born without hair and have to maintain a thermal balance in environmental temperatures equal to or less than 0 °C, so they must huddle in a nest that can provide them with a warm environment [39].

It is important to mention that one way to assess the thermogenic capacity of the BAT is through infrared thermography (IRT), a tool that has become very popular in recent years because it is a non-invasive technique, which provides measurements of superficial body temperature in a fairly accurate manner in dogs [40,41], ruminants [42], pigs [13], and rats [43,44,45]. Using IRT, Sokolof and Blumberg [44] demonstrated that the warmth provided by BAT appears to shape behavioral interactions, such as huddling in the group during hypothermia presentation. In another study, Marks et al. [43] observed that interscapular BAT does not seem to contribute to generating heat and does not appear to activate NST in rats. Moreover, weight is also a factor that influences thermoregulatory success. Lezama-García et al. [41] evaluated the thermal changes of 289 puppies during the first hours of life by taking thermal captures with the use of infrared thermography (IRT) when the newborns were wet, dry, colostrated, and 30 min, 1 h, 4 h, and 24 h after birth. The animals were divided according to weight into quartiles, with Q_1_ being the group with the lowest weight (126–226 g) and Q_4_ the group with the highest weight (388–452 g). The authors found a positive correlation between the weight of the pup and its success in thermostability for each body region evaluated, obtaining the highest temperatures in the central thermal windows and the lowest in the peripheral thermal windows, resulting in pups of the Q4 group having a better reported thermoregulatory capacity.

On the other hand, there are the precocial species capable of maintaining their core temperature (Tc) stable in extremely cold environmental conditions such as those mentioned above [46], and although they experience a rapid drop in temperature after birth, this forces them to increment of their heat production up to 15 times to make up for the loss [1,9,47].

Therefore, the generation of heat with and without shivering is recognized as some of the main mechanisms to deal with exposure to cold [48], using energy resources, or the activation of thermogenic systems [49], which will be explained below.

### 3.1. Non-Shivering Thermogenesis

NST is a method of heat production and attainment in cold environments [1,50]. The classic example of thermogenesis without shivering is that obtained from BAT, which is morphologically and metabolically different from white adipose tissue (WAT). For example, in sheep during the fetal stage, BAT appears in the perirenal region at 70 days of gestation [51], and in fact, among various mammalian species, the amount of BAT differs from 8 to 24 g, as does its triglyceride content (0.40–0.80 mg) [1,52].

It should be noted that BAT is a tissue that is not present in all species, as is the case in pigs [53] and wild boars (*Sus scrofa*) [14]. Likewise, and due to their size, small rodents such as mice and rats lack a functional BAT and do not use this thermoregulation mechanism efficiently. However, a second mechanism of NST has been described that is carried out in the muscle and is also known as muscle NST [14,54].

These different mechanisms of NST are discussed in detail in the subsequent sections.

#### 3.1.1. BAT as a Thermogenic Organ

This specialized thermogenic organ has a high content of cytochrome c that gives it its characteristic brown color, numerous mitochondria with many cristae, and small fat vacuoles, as well as abundant sympathetic innervation and blood supply [1,55,56,57,58,59].

**Figure 1:**
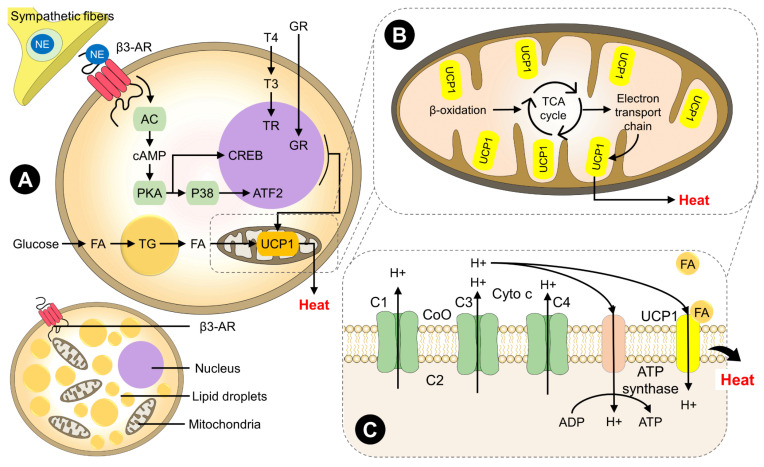
Characteristics and activation of the brown adipose tissue in mammals. When mammals are exposed to cold environments, it activates the sympathetic nervous system and the consequent release of catecholamines, notably NE from the adrenal glands. NE binds to β3-AR located in BAT to start a series of biochemical reactions to produce heat. cAMP production by AC results in the activation of the PKA, a protein that promotes lipolysis and thermogenesis through CREB, P38, and ATF2. Thyroid hormones (T4 and T3) also participate in gene expression and TG uptake, as well as GR and glucose. The conversion of TG to FA is used by the mitochondria to produce heat. In the mitochondria’s membrane, UCP1 receptors and cytochrome c participate in thermogenesis following β-oxidation, the TCA cycle, and the electron transport chain mechanism for thermogenesis. (**A**) Schematic deposit of BAT; AC: adenylyl cyclase; ATF2: activating transcription factor 2. (**B**) Mitochondria close-up; β3-AR: beta3-adrenergic receptor. (**C**) Mitochondrial membrane; cAMP: cyclic AMP; CREB: cAMP response element-binding protein; GR: glucocorticoid; FA: fatty acids; NE: norepinephrine; PKA: protein kinase A; T3/T4: thyroid hormone; TG: triglyceride; UCP1: uncoupling protein 1.

Scientific evidence has shown that the mitochondria of adipocytes have abundant amounts of an uncoupling protein-1 in their inner membrane, UCP1, which uncouples the electron transport chain from the electron transport-linked phosphorylation or oxidative phosphorylation process (the process through which energy as ATP is generated) of ATP synthesis, allowing protons from mitochondria to run back without ATP being produced, mainly to the generation of heat as an end product, rather than stored energy (Figure 1) [1,55,60,61,62]. BAT is able to utilize both glucose and fatty acids in mitochondrial metabolism [63]. Iatropoulos and Williams [64] mentioned that BAT has 50 to 100 times more mitochondria than other types of adipose tissue, contributing to its thermogenic capacity. In addition, BAT contains several small lipid droplets (multilocular), unlike the WAT structure, which is characterized by a single, large lipid droplet and few mitochondria. Moreover, BAT is also densely innervated by the sympathetic nervous system (SNS) and highly vascularized [63]. The same authors reported the role of BAT over time in 6-week-old and 36-week-old rats by performing hematological and histopathological studies on BAT. The results indicated that BAT maintains its activity in younger and older animals, and the proliferation of brown adipocytes is steady (around 20.5 to 28.9% of the replicating factor) [64]. These characteristics make BAT a tissue that participates in thermoregulation but that can also regulate homeostasis in other tissue structures.

The heat produced through this mechanism and fatty acid oxidation [65,66,67] is dispersed around the body through the bloodstream, ensuring that the core temperature is maintained within a narrow range after exposure to cold [67,68,69,70,71].

Canon et al. [72] and Gunn and Gluckman [73] mentioned that the thermogenic capacity of this tissue depends on the amount of UCP in the BAT and that it has been detected in lambs since the beginning of the third trimester of pregnancy [74]. Liu et al. [28] studied perirenal adipose tissue of newborn goats exposed to 6 °C for 24 h to evaluate its effects on gene expression and protein levels of UCP1. The results indicated that animals under 6 °C reduced their amounts of perirenal BAT pads and significantly increased UCP1 expression and triglycerides from 1-fold to around 0.8-fold. This represents the adaptative thermogenic response in goats, which is dependent on lipid contents in BAT. It is worth mentioning that UCP2 and UCP3 have been recognized in white and brown adipose tissue, as well as in muscle, but their roles in thermoregulation have not yet been fully elucidated [14,75].

The development of thermoregulation mechanisms involves a neurophysiological mechanism that has been widely studied in adult species and small mammals. Angilleta et al. [76] mentioned that rats (*Rattus norvegicus*) have a complex pathway for neuronal control of temperature since they have transient receptor potential channels in the skin, through which they send information about temperature to neurons in the dorsal horn of the spinal cord [77,78,79]. Subsequently, the dorsal horn, through afferent nerve fibers, transmits information to the lateral parabrachial nucleus at the brain stem level, specifically in the pons of Varolio [16]. Through the external lateral subregion of the lateral parabrachial nucleus the signal synapses with neurons of the preoptic area in the anterior hypothalamus and dorsomedial hypothalamus [80,81,82,83]. In the hypothalamus, the sympathetic and motor thermoregulatory responses are initiated [84].

**Figure 2:**
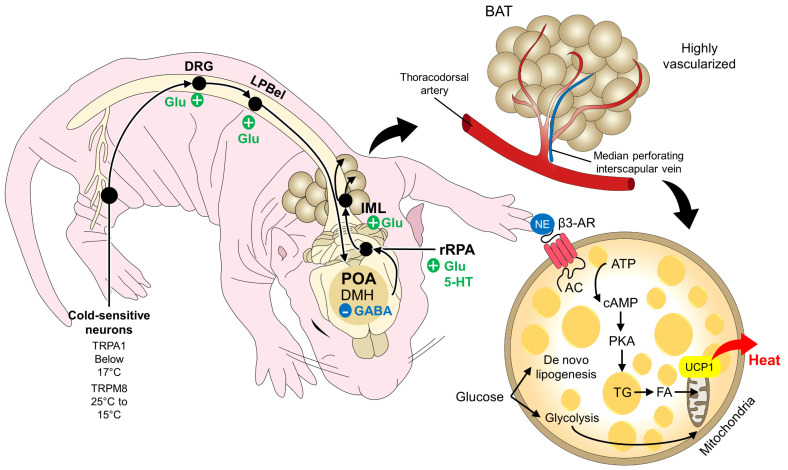
Non-shivering thermogenesis in neonate mammals. The activation of BAT to produce heat starts with the recognition of an environmental or core temperature drop by cold-sensitive neurons. The signals are transmitted to the DRG and the DMH in the POA by the LPBel pathway. From the DMH, a region where GABAergic neurons participate, projection to the rRPA reaches the IML region of the spinal cord. From this region, sympathetic afferents are directly connected to the BAT, where the release of NE activates the β3-AR thanks to the high vascularization present in BAT (through the thoracodorsal artery and the median perforating interscapular vein). The binding of NE to the β3-AR promotes lipogenesis, glycolysis, and the production of TG and FA as a fuel to the mitochondria to produce heat and increase core temperature. 5-HT: serotonin; AC: adenylyl cyclase; β3-AR: beta3-adrenergic receptor; cAMP: cyclic AMP; DMH: dorsomedial hypothalamus; DRG: dorsal root ganglion; FA: fatty acids; Glu: glutamate; IML: intermediolateral; LPBel: external lateral region of the lateral parabrachial nucleus; NE: norepinephrine; PKA: protein kinase A; POA: preoptic area; rRPA: rostral raphe pallidus; TG: triglyceride; UCP1: uncoupling protein 1.

In the case of the BAT thermogenic response, the cold signal in the hypothalamus stimulates the neurons of the median preoptic nucleus (MnPO), which inhibit the activity of the neurons of the medial preoptic area (MPA), which suppresses the activity of BAT thermogenesis promoting neurons present in the dorsomedial hypothalamic nucleus (DMH), thus allowing the activation of said neurons [85]. Subsequently, glutamatergic signals activate the BAT sympathetic premotor neurons located in the raphe pallidus area (RPA), which transmit this impulse to the intermediolateral cell column (IML) of the spinal cord: norepinephrine is finally released at the nerve endings. Sympathetic cells that innervate the BAT and activate β_3_ adrenergic receptors for lipolysis activate UCP1 so that oxidative phosphorylation is uncoupled to produce heat (Figure 2) [60,73,86]. In the naked mole rat, it has been reported that norepinephrine injection increases BAT temperature by 1.2 °C [87].

#### 3.1.2. Muscle Non-Shivering Thermogenesis

On the other hand, muscle NST begins with the activity of the Ca^2+^-ATPase pump in the sarcoplasmic reticulum (SERCA). SERCA1a, the principal isoform present in skeletal muscle, is generally involved in muscle contraction through the transport of Ca^2+^ ions from the cell lumen to the sarcoplasmic reticulum [88,89,90]. On the other hand, the presence of sarcolipin (SLN), a regulatory protein, can uncouple ATP hydrolysis (carried out by SERCA1a) from transmembrane Ca^2+^ transport, causing these ions to be released to the cytoplasmic in place of the membrane instead of the sarcoplasmic reticulum, resulting in an increase in ATP hydrolysis and heat production in the muscle without actual Ca^2+^ transport and thus without muscle contraction [91,92,93,94].

To elucidate whether muscle NST constitutes one of the most important and effective mechanisms for generating heat in wild mammals that lack BAT, Nowack et al. [14] developed a study in which they exposed wild boar piglets to cold (10 °C) for 60–90 min after their birth (day 1) and four days after that (day 5) determined heat production, weight gain, rectal temperature, and shivering intensity, as well as SERCA ATPase activity and SLN and SERCA1a gene expression. The results indicated that heat production did not differ significantly between days (day 1: 11 watt/kg; day 5: 12 watt/kg), nor did body temperature (day 1: 38.6 ± 0.09 °C; day 5: 39.0 ± 0.07 °C), in addition, it was detected that the activity of SERCA, as well as the expression of mRNA of SERCA1a and SLN, grew significantly from the first day to the fifth.

Nowack et al. [14] evidenced the contribution of muscle NST to heat production in wild boar piglets exposed to cold in their first days of life, indicating that shivering did not influence as much as muscle NST. From the above, it can be inferred that muscle NST is the main process of heat production in mammals that lack BAT or do not possess a functional UCP1, the main protein responsible for thermogenesis in the adipose tissue. It is worth mentioning that in rats and mice, species for which the participation of both muscle NST and BAT has been described, it has been detected that at normal temperature (23 °C) there is an elevated transcription and translation of SLN after birth, which thereafter reduces as BAT is recruited [67,90,95]. Nevertheless, when faced with cold environmental conditions, the mice maintain the SLN regulated for a better thermoregulatory capacity [95], an aspect that would lead to the conclusion that both NST mechanisms are necessary to maintain a high body temperature in newborn rodents exposed to cold [14].

### 3.2. Shivering Thermogenesis

**Figure 3:**
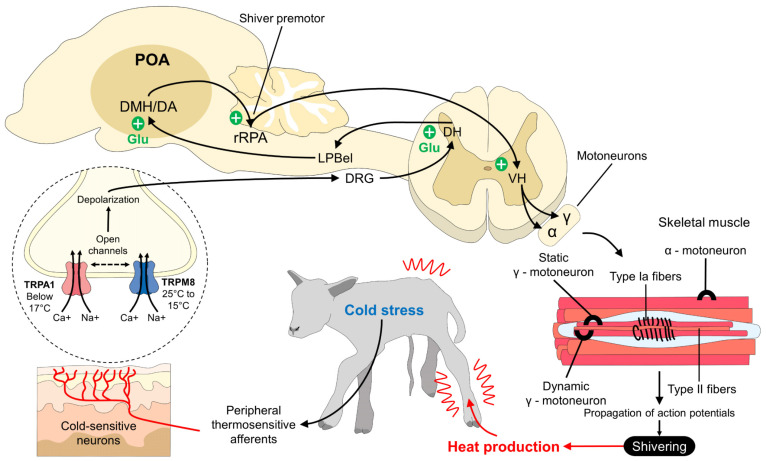
Neural circuit of shivering thermogenesis. When exposed to cold stress, peripheral thermosensitive afferents (known as cold-sensitive neurons) TRPA1 and TRPM8 detect noxious cold. The stimulus is transduced into an electric signal to ascend through the DRG and DH to the hypothalamus POA using the LPBel. In this pathway, glutamatergic neurons have an excitatory role in the premotor shiver nucleus located in the rRPA. Subsequently, these fibers stimulate the VH and α and γ motoneurons that innervate the skeletal muscle and, when excited, contract striated muscle fibers. The afferent input reaches type Ia and II fibers that enhance muscle fiber action potential to produce shivering and, consequently, the heat production. DA: dorsal hypothalamic area; DH: dorsal horn; DMH: dorsomedial hypothalamus; DRG: dorsal root ganglion; Glu: glutamate; LPBel: external lateral part of the lateral parabrachial nucleus; POA: preoptic area; rRPA: rostral raphe pallidus; TRPA: transient receptor potential ankyrin 1; TRPM8: transient receptor potential melastatin 8; VH: ventral horn.

Within the thermogenic responses of the neonate to external environments with low temperatures, shivering is observed; this comes from the production of heat by shivering skeletal muscles where, even though the neonatal muscles and blood supply are immature [3], heat generation is achieved through the repetitive and constant action of muscle contraction (Figure 3). During this procedure, oxygen consumption and the anaerobic capacity of muscle fibers increase, leading to the oxidation of energy molecules such as carbohydrates and fatty acids [34,96,97].

It has been reported that shivering contributes 60% to the thermogenesis of newborn lambs [98]. The canine neonate has a low capacity to perform thermogenesis with tremors because it uses other physiological mechanisms, such as respiratory and cardiovascular pattern changes. Due to all of the above, neonatal monitoring and evaluation are necessary to minimize the risk of mortality, since these adaptations include a stable and functional thermoregulatory mechanism until after the first four weeks of age [99].

On the other hand, for precocial species (such as the suckling pig), a greater susceptibility to cold is observed due to their poor insulation and deficient energy reserves, observing thermogenesis with shivering from the first two postnatal days. Despite what has been previously said, it is considered that shivering thermogenesis does not play a fundamental role like NST [56]; scientific evidence indicates that the energy source used for this thermogenesis mechanism generates high metabolic rates, such as newborns lambs is mostly fat, and represents 2% of their body weight [100,101].

Berthon et al. [102] studied the response of Large White neonatal piglets exposed to a thermoneutral (34 °C) and cold (25 °C) environment during the first 6 h up to 54 h of life; they observed metabolic changes, thermal changes, and heat production by shivering thermogenesis. The most relevant results indicated that piglets exposed to cold generated more heat (32.1 ± 0.9 vs. 19.8 ± 0.7 kJ/h/kg of body weight) and had a higher shivering activity (121.3 ± 25.4 vs. 4.6 ± 1.4 mV /min in piglets exposed to 34 °C). Both aspects were related to a reduction in muscle glycogen (−47%) and lipid content (−19 and 27% in the rhomboideus and longissimus dorsi muscles, respectively), reporting a reduction in lactate levels (−43%) and better muscle cytochrome oxidase activity (+20%). This confirmed that piglets develop shivering capabilities during short-term exposure to cold, being their main thermogenic mechanism [103].

With the above, the possibility of the development of NST is ruled out in piglets, since the mitochondria isolated from their muscles exposed to cold were not loose-coupled, in addition to the fact that the literature indicates that several weeks of cold acclimation are required for their development. Jansky [104] suggested an inadequate development of the temperature control systems at birth in this species [102].

## 4. Intrinsic Factors Involved in the Generation of Heat through BAT

### 4.1. Physiological Factors

The development of BAT originated during embryogenesis and is commonly located in the interscapular, perirenal, and perivascular areas through a series of cellular signaling from the dermomyotome [63]. There is a differentiation in the BAT in terms of the classic or constitutive type that develops in the fetus (at the interscapular area) during pregnancy, and the recruiting BAT or beige or brite adipose tissue, which is incorporated into the WAT deposits and the musculoskeletal system [16]. This process is known as WAT browning and gives the ability to express amounts of UCP 1 similar to the constitutive BAT as well as participate in thermogenesis [105].

Thermogenic capacity is defined as the activation of the cell to increase oxidation levels through cellular respiration. There are two types of thermogenic population in the BAT: high thermogenic brown adipocytes (BA-H), which have high expression of UCP1 as well as high mitochondrial populations with a round shape, and low thermogenic brown adipocytes (BA-L). These have little expression of UCP 1, low mitochondrial content with an oval shape, and intermediate-level mitochondrial respiration [106].

### 4.2. Biochemical Factors

BAT is activated in response to exposure to cold; processes that involve thyroid hormone, such as the local conversion of tetraiodothyronine (T4) to triiodothyronine (T3); and the increase in plasma cortisol during labor through the release of norepinephrine by the sympathetic innervation [19], increasing the levels of cyclic adenosine monophosphate (cAMP). This increase causes lipolysis of intracellular triacylglycerols (TAG) to convert them into free fatty acids (FFA) through protein kinase A (PKA) and initiate the beta-oxidation process through the production of acetyl coenzyme A (acetyl-CoA) and its oxidation to generate NADH and FADH_2_, triggering the proton gradient; this gradient disappears in the inner membrane of the mitochondria through the expression of UCP1 from the BAT, causing an increase in mitochondrial respiration and as a result the generation of heat [67,107].

During the perinatal period, the role of BAT in thermoregulation is also influenced by the availability of glucose and fatty acids, which are the main substrates for BAT thermogenesis. Glucose is derived mainly from glycogen stores and gluconeogenesis, whereas fatty acids are obtained from milk or adipose tissue lipolysis (triglycerides catabolism). However, during the perinatal period, glucose availability is limited and fatty acids become the primary substrate for BAT thermogenesis, brought through blood circulation. Of note, maternal undernutrition of species where placental transport of fatty acids is low (e.g., sheep and rats) results in a decrease in BAT deposition. This effect can be enhanced by a reduction in placental size or its ability to transport glucose during maternal undernutrition [108].

In addition to endocrine factors (thyroid hormones and catecholamines), some other elements and structures can promote BAT function, for example, the liver, since it has been observed that the postnatal maturation of BAT in rats is related to the beginning of food intake and the beginning of the liver function, which is mediated by the release of growth factor from fibroblast growth factor 21, a factor that may also promote BAT function [109] and appears to promote browning of some white adipose tissue deposits [110]. However, deletion of the BAT-derived fibroblast growth factor 21 does not alter BAT gene expression, which would lead to the conclusion that it actually plays a modest role in energy balance [110]. In neonates, the placenta is rich in deiodinase, leading to deiodination hormones [111,112] in the circulation, which are essential for the initiation of said thermogenic response [56]. Some studies carried out in lamb fetuses that were surrounded with a plastic coil in which cold water was circulated to provoke thermogenesis without shivering determined that no changes in the levels of free fatty acids and glycerol or increases in the temperature of brown fat were observed. These results led them to conclude that, although there is a marked increase in blood flow to brown fat [113], this thermogenic response does not appear to develop in the brown fat uterus due to the large amounts of oxygen that it requires [2], or due to the lack of catecholamines [114].

### 4.3. Molecular Factors

BAT is found mainly in the nuchal subcutaneous tissue, thoracic region, interscapular region, axillae, mediastinum, inguinal region, and around the spinal cord and kidneys [1,55,56,115]. Brown fat adipocytes located in different anatomical locations arise from distinct developmental origins: mesenchymal stem cells differentiate into dermomyotomal progenitors and give rise to constitutive brown adipocytes in the interscapular area, whereas mesodermal progenitors produce inducible brown adipocytes (or brite adipocytes) in WAT and muscles [63]. In rodents, apart from the classic BAT that arises from mfy5 (a kind of muscle-like cell lineage whose conversion to brown adipocytes is regulated by bone morphogenetic protein 7 [116,117]), white and brown adipose tissue that does not derive from a myf5-positive lineage has been described [118]. Differences in the development of this tissue are mediated to some extent by changes in thyroid function, which is critical for the initiation of BAT thermogenesis in both large and small mammals [119,120]. Part of this is due to triiodothyronine having the ability to serve as a bipotential mediator of mitochondrial biogenesis [121].

In addition, it is known that the rapid increase in UCP1 near birth depends on the rapid appearance of endocrine stimulatory factors such as cortisol, prolactin, leptin, and catecholamines [122]. In turn, the secretion of these factors will depend on the stress during birth, since this leads to intense activation of the central nervous system [123]. However, the magnitude of the response will depend on the exposure of the neonate to the cold of the extrauterine environment [2,110] and is highly influenced by individual characteristics based on the species.

### 4.4. Genetic Factors That Regulate the Presence of BAT

Certain genetic and phenotypic factors influence the thermogenesis of animals and the presence of BAT in neonates [1]. Among these factors, early or late development at birth has a high influence on thermoregulation [124]. In the first instance, altricial species are classified as immature animals at birth with a limited thermoregulatory capacity, requiring permanent maternal care during the first post-natal weeks [125]. Examples of altricial mammals are rodents, dogs, cats, monotremes, and marsupials [126,127]. Precocial neonates, such as ungulates, equines, and guinea pigs, are born with a mature HPA axis and a competent thermoregulatory system [34,128].

Symonds et al. [128] mention that BAT maturity differs between altricial and precocial animals even before birth. In rat pups, BAT maturity starts at birth and gradually increases during the first 10–20 days [129], culminating approximately 7 days after delivery, when the HPA becomes fully functional [72,110]. In contrast, in precocial species such as lambs, maturity of BAT occurs 40 days before birth, peaks at birth, and rapidly decreases in the first 10 post-natal days. In this species, birth is also accompanied by an increase in voluntary muscle activity and the onset of shivering thermogenesis, a response depending on the BAT function and the magnitude of the thermal challenge [110,130,131]. Cannon and Needergaard [132] mentioned that the biochemical thermogenic index of BAT (known as the total amount of UCP1 per body weight) and its physiological capacity (the increase in O_2_ after noradrenaline administration) are events that follow the same pattern in precocial and altricial species, suggesting that in altricial neonates BAT recruitment might be an adaptive response to extrauterine life that triggers the development of BAT during the first days after birth.

**Figure 4:**
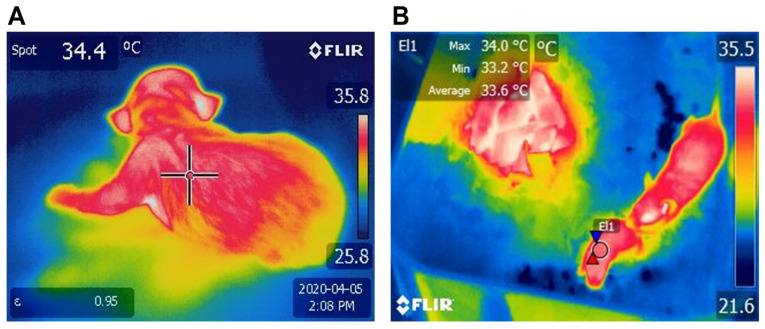
Radiometric images showing temperature differences in areas with brown adipose tissue of altricial neonates. (**A**) Chihuahua puppy. In dogs, BAT pads are in the interscapular, perirenal, and pericardial regions. Evaluating the surface temperature of BAT (spot) shows an average value of 34.4 °C. (**B**). Wistar rat pup. When comparing the average temperature of the interscapular BAT (El1) of the rat pup (33.6 °C) with the puppies, there is a difference of 0.8 °C. This difference can be attributed to the difference in size and the presence of hair at birth in dogs, whereas rat pups are born hairless and their body surface area is larger than their overall size, losing a greater proportion of heat. Maximal temperature is indicated with a red triangle and the minimal with a blue triangle. Radiometric images were obtained using a T1020 FLIR thermal camera. Image resolution 1024 × 768; up to 3.1 MP with UltraMax. FLIR Systems, Inc. Wilsonville, OR, USA.

To address specific changes based on the species, in the case of altricial animals, several studies have been performed on rodents. In rat pups evaluated during the first 20 h after birth, the amount of UCP’s mRNA increased to 152 ± 19 µg within 2 h. The results of this study also reported a gradual decrease in mRNA that corresponded to the increase in body weight, meaning that animals weighing 5.86 ± 0.12 g had the highest amount of UCP mRNA and pups weighing 6.00 ± 0.18 g had the lowest values (95 ± 11 µg) [133]. In the case of Syrian hamsters (considered a highly immature neonate), BAT development occurs until post-natal day 16 and continues during the lactation period, making them susceptible to temperature changes after birth [129,134]. A similar response can be observed in Figure 4, where a comparison between newborn puppy dogs and pup rats was performed using IRT, where BAT average surface temperatures were higher in pups.

**Figure 5:**
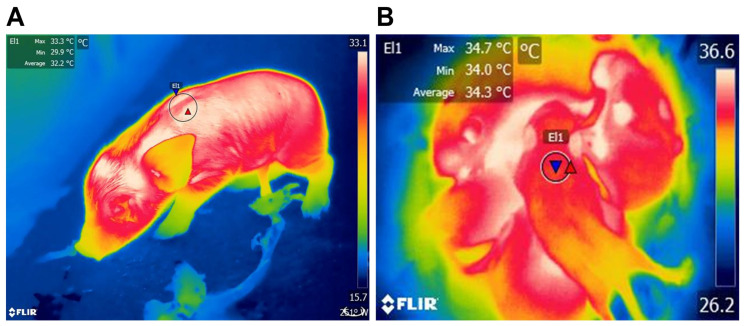
Temperature comparison of the interscapular region between species with and without BAT at birth. (**A**) Large White X Landrace newborn piglet. The maximum, minimum, and average temperature of the piglet at the interscapular region (El1) shows values of 33.3 °C, 29.9 °C, and 32.2 °C. (**B**) Wistar rat pups. The BAT temperature (El1) registered increases in the temperature of the pup when compared to the piglet’s information. The average temperature increased by 1.4 °C, whereas increases of 4.4 °C and 2.1 °C were recorded for the minimum and average temperatures. The differences in surface temperature between both species can be attributed to traits in each animal. Although pigs, a semi-altricial species, are born with sparsely distributed hair and are bigger than rat pups, they are born without BAT reserves, making them susceptible to hypothermia. In contrast, although rat pups are born hairless, they have interscapular BAT reserves that produce heat when exposed to cold stress and rely on behavioral modifications such as huddling to preserve heat. Maximal temperature is indicated with a red triangle and the minimal with a blue triangle. Radiometric images were obtained using a T1020 FLIR thermal camera. Image resolution 1024 × 768; up to 3.1 MP with UltraMax. FLIR Systems, Inc. Wilsonville, OR, USA.

In puppy dogs, an altricial species, the daily pattern of the oscillations in body temperature becomes evident from the fourth week of life, similar to that reported in human infants [40]. In mice, interscapular BAT deposits appear on embryonic day 15.5 [135] and increase by nine times from birth to pubertal age. In this species, a study aimed to understand the post-natal proliferation and lipogenesis of BAT showed that increasing the size and number of brown adipocytes is the main event in the first two weeks of life, whereas enlargement occurs in the following days, significantly increasing BAT’s weight by 11 times within 30 days [136]. These studies exemplify the importance of post-natal development of BAT in altricial species, an element that is closely related to behavioral thermoregulatory responses in immature animals at birth, such as huddling or nesting, which are critical to preserving heat [34]. A comparative example of this is shown in Figure 5, where a semi’s/altricial’s development at birth is compared to the thermoregulatory traits of rat pups.

In contrast, in precocial animals such as lambs, the amount of perirenal BAT mRNA, activity of the UCP, mitochondrial protein, and lipid content during the first month of life depends on body core temperature, with the largest amount of UCP mRNA found at four to seven days after lambing [137,138]. However, it has been reported that although a lamb’s BAT contributes approximately 50% of thermogenesis at birth, this response is diminished at one month old, when there is not a significant mechanism to thermoregulate [1,139]. In Hereford x Friesian calves, Alexander et al. [140] reported that BAT corresponds to approximately 2% of the body weight of newborn calves and that adipose tissue present in the omental, mesenteric, intestinal, abdominal, cervical, pericardial, cardiac, groove, perirenal, prescapular, popliteal, and orbital regions had brown adipocytes.

Apart from altricial and precocial differences, the natural environment and characteristics of the species also influence the amount or presence of BAT. Pigs can be considered semi-altricial animals, since piglets are born with functional systems and sensorial organs but cannot thermoregulate properly immediately after birth, and sows form a nest to prevent heat losses within the litter [13,141]. The susceptibility to cold stress is due to the apparent lack of BAT in piglets, who use shivering to produce heat. Authors such as Berg et al. [142] mentioned that pigs have mutations for the UCP1 coding sequence, inactivating the thermogenic capacity of the receptor and, therefore, impeding NST. Contrarily, in harp seal pups, animals that are highly susceptible to heat loss due to the lack of subcutaneous blubber and infantile fur with poor insulation capability, Grav and Blix [143] mentioned that NST through subcutaneous BAT activation is critical to newborn seals immersed in ice water, reporting that subcutaneous temperature was similar to body core temperature, meaning the sympathetic activation of BAT. Genetic studies in lambs indicate that neonatal thermogenesis depends on the breed’s characteristics [144]. For example, in Scottish blackface lambs, the resistance to low temperatures could be heritable by 30% [145], an estimate like that obtained in crossbred lambs [146]. Similarly, in a comparative study infusing NE to Wagyu x Angus, Angus, and Brahman’s calves, Smith et al. [147] described the differences in metabolism, location, and thermogenic capacity of BAT. Among the breeds, during the fetal stage, all presented UCP1 gene expression. However, although Brahman’s calves had more β-receptors and lipogenic activity than Angus calves, Brahman’s calf BAT shrank after cold exposure, representing lipid exhaustion in the breed. The relevance of identifying these differences among breeds is that greater neonatal mortalities have been reported in Brahman calves exposed to cold, suggesting that UCP activity and UCP1 mRNA of the subcutaneous BAT, which is greater in Angus neonates, could be involved in their thermogenic efficacy, as stated by Landis et al. [148].

Regarding phenotypic factors, body weight, coat, and skin properties also influence the resistance to cold. Studies in species such as puppy dogs [40,41], buffalo calves [149], piglets [150,151], lambs [152], and dairy calves [153] have shown an association between birth weight and thermoregulatory capacity. In lambs, heavier newborns have a lower surface-to-volume ratio, which implies a lower tendency to lose heat, having higher energy reserves to thermoregulate [1,154].

In the case of dogs, Piccione et al. [154] compared pups from three different breeds (Rottweiler, n = 7; Cocker Spaniel, n = 5; Carlino, n = 4) to determine the influence of the circadian rhythm with body temperature rhythm (measured with rectal temperature for 2 months just after birth) and growth rate. The authors reported a progressive increase in rectal temperature between 0.6 °C in Carlino and 1 °C in Rottweilers, and differences in birth size and birth weight, with Carlino puppies having lower values. However, no relation between the circadian rhythm and high temperature was observed. This increase is a phenomenon described in several species, reflecting that body heat loss decreases over time due to a lower body surface–body mass ratio in older animals [124].

**Figure 6:**
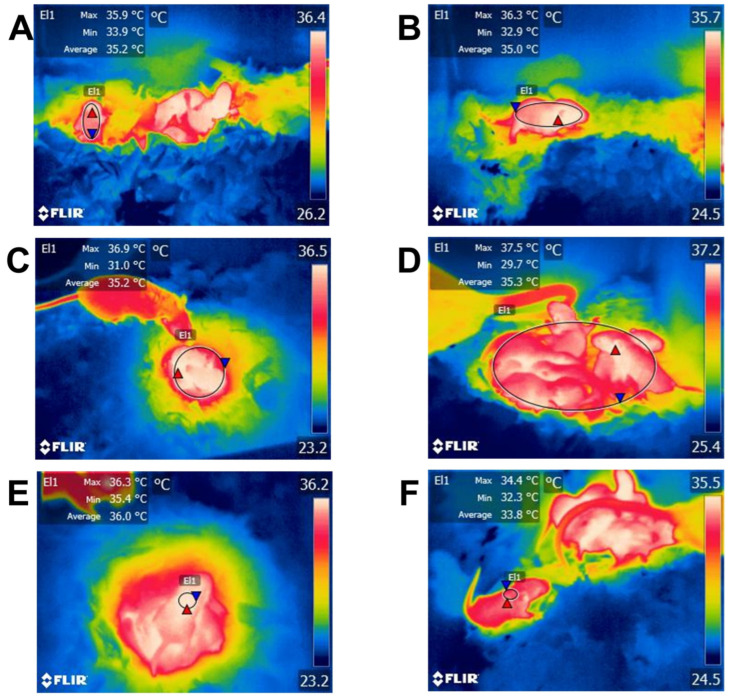
Influence of hair and post-natal days on the newborn rat pup. (**A**,**C**,**E**) thermograms show hairless 1-day-old rat pups, whereas (**B**,**D**,**F**) show 1-week-old rat pups with hair. (**A**,**B**) When comparing the maximum temperature between a hairless ((**A**), El1) and a 1-week-old rat pup ((**B**), El1), the temperature of the pup with hair is 0.4 °C higher. (**C**,**D**) The maximum temperature of the nest of newborn rat pups ((**C**), El1) is 36.9 °C, whereas that of the pups with hair ((**D**), El1) is 37.5 °C, 0.6 °C higher. (**E**,**F**) Regarding the position of the neonate in the nest, it can be observed that 1-day-old pups stay inside the nest, next to their littermates, with a maximum BAT temperature ((**E**), El1) of 36.3 °C. In contrast, the BAT temperature ((**F**), El1) of the pup with hair is 1.9 °C lower than the (**E**) pup. In general, it can be observed that 1-week-old rat pups have thermoregulatory benefits due to the presence of hair and the increase in size, which make them auto-sufficient to thermoregulate and not dependent on nesting to maintain their body core temperature. The decrease in BAT temperature of older animals could also be explained by a higher thermoregulatory challenge in hairless neonates, where BAT requires more activation to produce heat. Maximal temperature is indicated with a red triangle and the minimal with a blue triangle. Radiometric images were obtained using a T1020 FLIR thermal camera. Image resolution 1024 × 768; up to 3.1 MP with UltraMax. FLIR Systems, Inc. Wilsonville, OR, USA.

Regarding the coat, it is known that characteristics such as length, color, type, and presence/absence influence the thermoregulatory mechanisms of animals even immediately after birth [155]. In lambs, a significant positive phenotypic correlation has been found between the depth of the coat and the resistance to cold [156]. On the other hand, the thickness of the lamb’s skin also seems to be involucrate in their capacity to tolerate cold temperatures in several breeds [144,157]. In this sense, lambs with less thin skin can compensate for hypothermia better because they have more subcutaneous fat deposits. However, Slee et al. [144] mentioned that lambs do not have as much subcutaneous fat but have managed to better compensate for heat loss because their peripheral blood vessels are slightly far from the external temperature. It should be noted that even after adjusting for weight and coat and skin properties, there are some lambs within a breed that are more resistant to hypothermia. In contrast, other breeds struggle to keep their temperature stable under cold conditions, which suggests the existence of physiological changes that can cause some breeds to respond better than others to these elements [1,158]. Preliminary studies conducted by the authors to assess the influence of hair in the thermoregulation of newborn rat pups showed that 1-week-old neonates have an increased thermoregulatory capacity when considering the surface temperature, as observed in Figure 6.

Finally, litter size associated with the type of species (altricial and precocial) is another factor related to cold resistance and BAT activation [157]. However, this has been reported in the dam, as mentioned by Isler et al. [159] in lactating rats, where the thermogenic activity (assessed by BAT mass and protein content) was fully suppressed in females nursing large litters (12 pups). Thus, studies evaluating the effect of litter size are required in neonates. An example was provided by Xiao et al. [160], who found that rats from small litters (3 pups) exposed to overnutrition during the early postnatal period (from birth to weaning) experienced permanent changes in the function and development of their BAT compared to rats from a normal litter size (8 pups). Specifically, the rats had decreased BAT thermogenic capacity (lower levels of UCP1), increased insulin resistance, and altered gene expression patterns (notably involved in the SNS activity) compared to control rats (normal litters) that were not exposed to overnutrition. Likewise, de Almeida et al. [161] found that overfed rats (from a small litter size: 3 pups) had lower levels of BAT activity than rats who were not overfed (from a normal litter size: 9 pups), lower thermogenic capacity, and adipocyte morphological differences with fewer lipid droplets. This reduced BAT activity may contribute to the development of obesity, as BAT plays a role in regulating energy balance and thermogenesis. The authors also looked at the molecular mechanisms underlying the reduced BAT activity and found that it was associated with changes in the expression of genes involved in BAT function and metabolism. Regarding metabolic function, another study reported that litter size reduction induced metabolic changes in the dams, including increased food intake, decreased insulin sensitivity, and altered lipid metabolism. The authors also observed histological changes in the mammary glands of the dams, which may have implications for milk production and nutrient delivery to the offspring. The authors further investigated the effects of litter size reduction on offspring growth and development. They found that offspring SL displayed higher body weight and growth, hyperglycemia, augmented lipid deposition in BAT, and elevated islet. Overall, that paper demonstrated that litter size reduction induces metabolic and histological adjustments in lactating dams, which have early effects on offspring growth, metabolism, and development [162].

BAT remodeling and phenotypic flexibility is another phenomenon that occurs through generations of animals exposed to cold stress during critical developmental stages and their reaction to the same stressor in adult life. In *Peromyscus maniculatus*, mice pups born from cold-exposed mothers during pregnancy (14 °C) showed a limited capacity to adjust the thermogenic mechanism, whereas pups reared at 24 °C increased their metabolic rate earlier than control pups, and the mass of BAT increased at post-natal day six [163]. Besides, Symonds and Lomax wrote that cold stress during late pregnancy stimulates the capacity of newborn lambs and rats, also improving the survival of the neonate [164].

Most of the studies addressing BAT activation and thermogenesis used environmental factors as a stimulus to understand the genetic differences that species present. Therefore, it is important to know and understand the association between intrinsic and extrinsic factors that can trigger NST through BAT.

## 5. Extrinsic Factors Involved

Within the extrinsic factors involved in the management of hypothermia in newborns, we can mention the consumption of colostrum, the administration of warm water orally in newborns, the route of delivery, the position in the litter, maternal food restriction, and maternal care.

### 5.1. Colostrum and Milk Consumption

In addition to providing an important contribution of immune passive factors, colostrum and milk are high sources of nutrients and energy to meet the demands of the neonate during the early postnatal period [165]. Nutrition is a key factor for neonates, due to the importance of glucose and fatty acid availability that is obtained through colostrum. In fact, lambs that have consumed colostrum have reported higher rectal temperatures, as well as higher levels of circulating glucose, which they have registered (up to 43% after being exposed to cold temperatures) [1,166]. Therefore, for lambs receiving proper feed, mortality rates decrease. In contrast, in animals with inadequate feeding, the incidence of mortality is linked to the limited energy reserves present during the first 3–5 days, a period that is reduced by cold [1,47,166,167]. Milk consumption seems to be vital for the initiation of BAT thermogenesis, even though its composition varies between species. Within the components that have been found in human milk, on the one hand, it is mentioned that this is characterized by having a high cortisol content [168], a very important endocrine stimulating factor of UCP1 when childbirth approaches, but, it has not yet been fully determined whether cortisol promotes the synthesis of these proteins in neonates to the same degree that has been observed in fetuses [169]. On the other hand, it is also reported that human milk has a high content of prolactin, which is involved in promoting the development of BAT and in ensuring that the thermogenesis of this tissue is maintained during postnatal development [170,171]. Therefore, its consumption could lead to greater BAT activity later in life, since only directly stimulating prolactin receptors (PRLR) promote BAT thermogenesis [172]. It has been reported that, at least in neonatal sheep, prolactin has a rapid thermogenic effect when administered intravenously. However, it is uncertain whether this response also occurs when prolactin is ingested [110,172]. Thermography has shown an important thermogenic effect of drinking human milk in young children, since after ingesting it, they presented an increase of 0.7 °C in BAT temperature [110]. The magnitude of this response seems to be given by the composition of macronutrients [173], with their fat composition peaking after birth but declining one week after delivery [110,174].

One aspect to consider is that the evidence indicates that the magnitude of BAT thermic responses after feeding seems to depend on the environmental temperature to which the lambs are exposed. When administering 50 mL of warm colostrum, the thermic compensatory activity of BAT only increased in lambs that were kept in an environment with a warm temperature, whereas a decrease was reported in those that were exposed to a temperature of 15 °C [175].

It should be noted that, at least in lambs, cooling suppresses body functions such as suckling [5,176], which can lead to starvation, which reduces the ability to maintain a high metabolic rate, and therefore animals become more susceptible to secondary hypothermia [47,176]. Other events to consider that may interfere with milk intake are udder and teat abnormalities, poor milk supply [1,149], and physiological maturity, and, at least in premature human infants, an impaired sucking reflex has been reported compared to born-at-term infants [177].

However, it is necessary to emphasize that even after consumption of milk, animals can present primary hypothermia due to exposure to environmental factors in the presence or absence of a complete metabolism of BAT [1,149].

### 5.2. Oral Administration of Warm Water to Newborns

One study showed that the fact of administering warm water to newborns can cause an increase in colonic temperature and in the concentrations of metabolites and catecholamines in the plasma, which varies depending on the environmental temperature in which the animals are kept. On the one hand, no effect of feeding on endocrine or metabolic status was observed in lambs that were kept in a warm environment, whereas in lambs exposed to a cold temperature, feeding caused an increment in plasma glucose concentration (before food: 6.89 ± 0.02 mM; after food: 7.35 ± 0.02 mM) and non-esterified fatty acids (before food: 0.66 ± 0.10 mM; after food: 0.95 ± 0.12 mM), together with a decrease in concentrations of adrenaline (before food: 3.9 ± 0.1 mg mL^−1^; after food: 1.2 ± 0.1 mg mL^−1^) and dopamine (before food: 5.0 ± 0.1 mg mL^−1^; after food: 2.7 ± 0.1 mg mL^−1^) in plasma. Likewise, lambs kept at a cold temperature presented a low concentration of adrenaline in their BAT (38 ± 46 vs. 144 ± 73 mg g^−1^ in the lambs exposed to 30 °C), contrary to what was reported in lambs that were fed with colostrum [75]. Finally, it was detected that the colonic temperature of the lambs exposed to a cold temperature increased after the administration of water (before food: 39.05 ± 0.10; after food: 39.55 ± 0.10), being that both groups had a similar amount of perirenal BAT (exposed to 30 °C: 21.5 ± 2.3 g; exposed to 15 °C: 19.7 ± 1.9 g) and mitochondrial protein (1563 ± 278 mg; CD: 1800 ± 357 mg), in addition to the fact that it was reported that this group presented a BAT with greater thermogenic activity despite having a lower type II 5′monodeiodinase activity (973 ± 202 vs. 1503 ± 292 in lambs exposed to 30 °C), whereas this enzyme catalyzes the conversion of T4 to T3 [73]. These findings indicate that the body temperature of lambs exposed to cold temperatures can be increased by the administration of only water, provided they possess sufficient BAT and other endogenous glucose reserves [178]. Comparing these results with those obtained by administering 50 mL of colostrum, it is concluded that environmental temperature does influence the thermoregulatory activity of BAT. Colostrum provides a greater thermogenic activity in lambs kept in a warm environment, more than changes in intestinal motility or hypothalamic activity associated with colostrum intake. However, it remains to be determined which of its nutrients are involved in these responses [75].

Similarly, the different mechanisms by which colostrum or water feeding may influence BAT activity and metabolism in neonates that may be exposed to different temperatures remain to be elucidated. Clarke and Symonds [75] developed a study that consisted of administering small volumes of hot water (25 °C) to 12 lambs at 5 h of age exposed to warm (30 °C) or cold (15 °C) to determine its effect on thermoregulation by measuring colonic temperature, metabolic (glucose, non-esterified fatty acids) responses, and endocrine (triiodothyronine, thyroxine, norepinephrine, adrenaline, dopamine) responses through blood samples, oxygen consumption, and CO_2_ production using indirect open-circuit calorimetry, as well as perirenal adipose tissue obtained after euthanasia of lambs. They point out that a possible explanation could be a change in plasma leptin concentration, which is known to increase sympathetic activity [179], which could be the link between diet and exercise. In adult ruminants, the abundance of mRNA for the leptin receptor in BAT is nutritionally regulated; therefore, providing good nutrition immediately after birth is critical [180]. However, it is urged to continue with the investigations to determine whether this also occurs in neonates and to know the degree to which temperature modulates leptin secretion.

To use these methods, it is important to compare the effect of different rearing temperatures with water at different temperatures to specify which stimulus maximizes BAT function [75]. Moreover, warm water administration might cause adverse effects to newborns when the temperature of the water is too high, causing burns and scalds, particularly in human infants [181]. In addition, although there are no studies in animals, in humans it has been reported that drinking warm (50–52 °C) or cold fluids alters evaporative heat loss by sweating and muscle activation, a change that can occur within one minute after warm water ingestion and might represent an increase in heat loss by sweating [182]. Nonetheless, further studies in animals are required to evaluate this effect.

### 5.3. Route of Parturition

Likewise, it is pointed out that the response is also affected by the route of parturition since the thermogenic activity is better in lambs delivered vaginally than in those born by cesarean section [31]. It was observed by these authors that colonic temperature was constant over the first 30 min of life in vaginally delivered lambs and lower in the group that was maintained at 30 °C, and in the animals that were born by cesarean section delivery colonic temperature rapidly decreased. They concluded that cesarean section delivery produces alterations in sympathetic, adrenal, and thyroidal activity, which appears to have a marked influence on BAT function, promoting many differences in thermoregulation compared with lambs born by vaginally lambing [31].

### 5.4. Maternal Food Restriction

The nutritional contribution of the mother during pregnancy is an extrinsic factor determining the development of the brown adipose tissue during pregnancy. A study was carried out in pregnant mice where a maternal food restriction of 50% of the common diet was performed, whose pups were sacrificed to quantify the expression of UPC 1, UCP 2, UCP 3, and SERCA 1. The results showed an increase in UPC 1 and UCP 2 production in response to the decrease in the amount of body mass and fat content of the pups, causing excessive heat loss as well as the inability to thermoregulate. However, there were no significant changes in the expression of UCP 3 and SERCA 1 [183].

In addition, in a study that involved feeding pregnant ewes either a control diet or a low-protein diet during mid-to-late gestation, Budge et al. [184] examined the fetal lambs to assess BAT development and PRLR expression. They found that maternal undernutrition reduced fetal BAT weight and altered PRLR expression in fetal BAT. Specifically, the low-protein diet led to a decrease in PRLR mRNA expression in fetal BAT. The study suggests that maternal undernutrition can have a significant impact on fetal BAT development and PRLR expression. On the contrary, in neonatal rats, increasing maternal protein intake during pregnancy is associated with both enhanced thermogenic activity in BAT and larger BAT depots [185]. In newborn rabbits, it has been observed that heavier rabbits from smaller litters are more likely to survive because they may have access to greater amounts of milk than larger litters and have more probability of surviving the critical first postnatal period. They also tend to occupy thermally advantageous central positions than lighter rabbits, same situation as in other mammals such as puppies or kittens [70,186,187,188,189,190,191].

### 5.5. Maternal Care

For all newborn mammals, the mother plays a very important role in their care and survival, since the newborn depends on her to receive heat, food, and the necessary care to survive, especially in altricial species during the first months of life [192]. Maternal behavior produces many hormonal and stress responsiveness pathways at the hypothalamic-pituitary-adrenal axis to help the neonate [193]. If the mother does not provide adequate maternal care, such as making the nest, licking, and bringing the newborns close to the teats to suck milk, they may have great difficulties in surviving, since they could die of starvation [194].

Maternal behaviors such as keeping close contact with newborns is a relevant element necessary to maintain thermal stability. Particularly in piglets, animals susceptible to hypothermia, when they are not nursing they spend between 10.6 and 16.1% of their time with their mother [195]. In the same species, maternal behaviors from week 15 of gestation to week 3 of lactation are associated with the thermoneutrality of both the sow and the piglet. During gestation, 40% of the sows stayed in the paddock with a wallow, whereas in the lactation period, the sows were seen in the huts (54%) [196]. These results suggest that sows can decide where to nurse the piglets according to the thermal environment. In the case of human newborns, maternal practices that include room warming, drying and covering with warm clothes, and exclusive breastfeeding prevent hypothermia [197].

### 5.6. Position in the Litter

As mentioned by García-Torres et al. [70], another aspect to consider in the development of BAT tissue is the position of the pups in the litter, since physiological, behavioral, and survival probability differences have been found among littermates since birth [191,198,199]. Studies in rabbits have found that heavier rabbit pups tend to stay in central positions inside the nest. This can be considered an energetic advantage because they spend less time climbing or pushing their peers to obtain food [70,186,187,188,189,190,191].

To assess this and determine whether there are individual differences in morpho-physiological parameters of BAT thermogenesis during acute and chronic drops in temperature exposure, García-Torres et al. [70] studied the thermogenesis of rabbit pups during the first 3 days after birth, which are the more elevated period of postnatal mortality [186,200,201], because their lack of fur and their high body surface to body mass ratio makes them particularly susceptible to hypothermia [189,202,203]. In their study, García-Torres et al. [70] divided 21 litters into 3 groups, each one made up of 7 litters. The first group (birth), made up of 63 young rabbits, was separated from their mothers at birth. The second group (chronic exposure to moderate cold), made up of 45 young rabbits, was placed in a cold room with the temperature set at 20 °C [70]. It has been reported that newborn rabbits reach a thermoneutral zone of 35 °C at days 2 and 3 post-birth [204,205,206,207,208,209]. Finally, group 3, made up of 43 young rabbits, was exposed to the same procedure as the second group, except that on day 3 the most central young rabbit (the one that had physical contact with a greater number of littermates during the 3 days) and the most peripheral (the one that had physical contact with the fewest number of littermates during the 3 days) pup of each litter were placed in ice boxes for 30 min to maintain the temperature between 9 and 11 °C. It is worth mentioning that in all groups the body mass was measured, and that in groups 2 and 3 milk intake and skin temperature were also quantified, specifically of the interscapular zone in the young rabbits from group 3 that were exposed to a temperature of 9–11 °C, with a thermographic camera. After the evaluation period, the rabbits from the three groups were sacrificed to perform a morphometric analysis of the left interscapular pad of BAT. In the same way, blood was collected from the trunk to later measure the concentrations of triglycerides in serum. The results suggested that in the first group, there was no significant difference in the number (84.1 ± 17.5 vs. 87.6 ± 20.7) or size (58.1 ± 7 vs. 60.8 ± 12.5 mm^2^) of the fat vacuoles, nor in the serum concentrations of triglycerides (118.7 ± 24.9 vs. 134.4 ± 38.5 mg/dL), which presented the smallest and biggest pups, respectively, at birth, indicating that there was no difference in the energy reserves of the pups, despite differing in weight [70].

**Figure 7:**
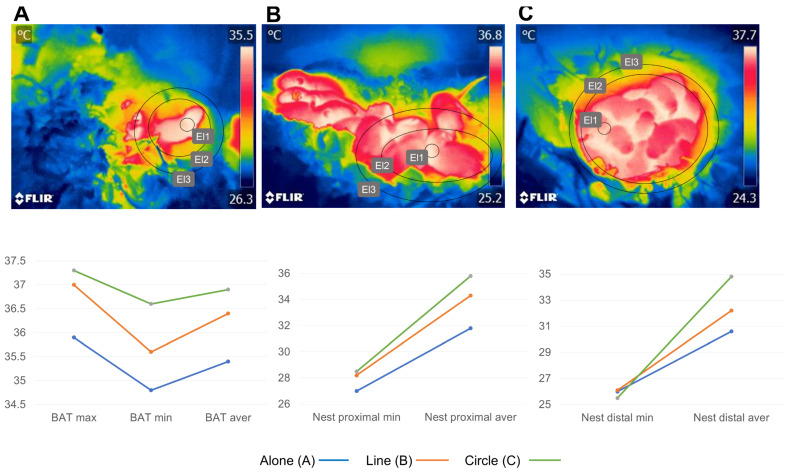
Importance of the position of the newborn in the nest for thermoregulation in 1-day-old Wistar rat pups. (**A**) Rat pups alone. The temperature of the interscapular BAT (El1) shows maximum, minimum, and average values of 35.9 °C, 34.8 °C, and 35.4 °C, respectively. When evaluating the proximal temperature of the nest (El2) around the pup, 26.0 °C is the minimum value recorded, the same as in the distal zone of the nest (El3). (**B**) Rat pups grouped in a line. The BAT temperature (El1) of one of the pups in this position has a maximum value of 37.0°, 1.1 °C higher than that of the rat pup alone. The temperature increases were also observed for the minimum and average values, being 0.8 °C and 1 °C higher, respectively, than the A pup. When comparing the minimum temperature of the proximal nest, a rise in temperature of 1.2 °C can also be observed (**C**). Rat pups huddling in a circle. This position promotes a maximum BAT temperature of 37.3 °C, 1.4 °C higher than that of the pup standing alone. Similarly, the minimal and average values of the proximal nest, when compared to the pup alone, are 1.5 °C and 4 °C higher, respectively. The high values observed in the pups formed in a circle are due to the importance of huddling, the most important behavioral adaptation of newborn rodents to prevent heat losses by maintaining contact with their conspecifics.

Results obtained in a preliminary study of the authors of the present review in Wistar rat pups show that, in general, littermates placed at the center of the nest have the highest temperatures, and the position of the animals (alone, in line, or in a circle) highly influences the amount of heat transmission (Figure 7). Interestingly, although huddling and nesting are considered critical behaviors to prevent heat loss in rodents, in the naked mole rat, Oiwa et al. [87] reported that rats can maintain their temperature whether isolated or in a group and that activation of BAT can also occur at 30 °C. Isolated animals had a maximum cervix surface temperature of approximately 34 °C, a similar value to the animals staying at a colony.

## 6. Future Directions

It is important to consider all current information to determine how these issues could improve both production and animal welfare, as well as decrease high mortality in some species. In the same way, it is essential to identify critical factors in the newborn to avoid them in a timely manner, as well as detect whether some breeds could be bred more easily in certain climates so the amount of BAT can aid in cold tolerance.

It is necessary to apply experimental models and evaluate through IRT, biochemical, and electrophysiological tests the effect of pharmacological treatments, surgical procedures, and behavioral tests and how this affects BAT thermogenesis. For example, there is evidence that pups from rats receiving prolactin during gestation have an increased amount of UCP1, but post-natal administration does not influence BAT elements (UCP1 and cytochrome c abundance) [210]. The same prenatal protocol could be tested in other species susceptible to hypothermia to study its efficacy as a preventive treatment. Moreover, specific treatments or management could be provided to the species or breeds in which BAT is lacking or is not as effective as conspecifics [148]. This would be an approach to prevent neonatal mortality due to hypothermia [6,149,211,212].

Likewise, future research must consider the metabolic processes involved in obtaining and using energy, focusing on the impact of nutrition. Moreover, the genetic selection of animals with a high thermal efficiency index and those with improved energy metabolism could help to minimize mortality rates due to hypothermia, the emission of greenhouse gases, and the carbon footprint generated by farm animals [213,214,215].

## 7. Conclusions

Birth represents a thermal challenge for neonates, so the initiation of thermogenesis is crucial to their survival, particularly in the first 36 h after birth, in the case of precocial species, or in the first 3 days after birth, in the case of altricial species, which is when the mortality rate is the highest. For this, newborns develop responses such as thermogenesis with shivering and NST, which involves the metabolism of BAT. However, not all species use these mechanisms to the same extent. Species such as lambs have BAT thermogenesis as their main mechanism, whereas piglets, devoid of BAT, present shivering thermogenesis from the first two days of life, although it should be noted that shivering in lambs goes from contributing 60% to thermogenesis in newborns to contributing 95% at 30 days of age. Contrary to the above, it has been detected that in wild boar piglet muscle NST constitutes an important mechanism that, over time, replaces the percentage contribution of shivering. In the case of newborn rodents, evidence suggests that both mechanisms of NST (muscular and BAT) are necessary to maintain elevated body temperature in the face of cold environmental temperatures, which demonstrates the diversity of thermogenic responses that species can present. On the other hand, the existence of a great variety of intrinsic and extrinsic factors is described that can influence the thermogenic responses of neonates of different species.

However, all the knowledge that is available up to now can be useful to define when to intervene to ensure the thermoregulation and survival of the neonate, especially if it is premature; in what way it could be done; and for how long (considering that atricial species take longer to achieve thermoregulation on their own), because even though neonates have thermoregulation mechanisms, the vast majority of them are not fully functioning immediately after delivery. Through this, it will be possible to limit one of the main causes of mortality in neonates, hypothermia, which will bring benefits for both animals and producers.

## Data Availability

Data sharing not applicable.
